# Fast Quantification of Honey Adulteration with Laser-Induced Breakdown Spectroscopy and Chemometric Methods

**DOI:** 10.3390/foods9030341

**Published:** 2020-03-14

**Authors:** Jiyu Peng, Weiyue Xie, Jiandong Jiang, Zhangfeng Zhao, Fei Zhou, Fei Liu

**Affiliations:** 1 Key Laboratory of E & M (Zhejiang University of Technology), Ministry of Education & Zhejiang Province, Hangzhou 310014, China; jypeng@zjut.edu.cn (J.P.); wyxiee@163.com (W.X.); jiangjd@zjut.edu.cn (J.J.); i12fly@163.com (Z.Z.); 2 College of Standardization, China Jiliang University, Hangzhou 310018, China; 3 College of Biosystems Engineering and Food Science, Zhejiang University, Hangzhou 310058, China; fliu@zju.edu.cn; 4 Beingmate (Hangzhou) Food Research Institute Co., Ltd, Hangzhou 311106, China

**Keywords:** honey, adulteration, feature variable, partial least square regression, laser-induced breakdown spectroscopy

## Abstract

Honey adulteration is a major issue in food production, which may reduce the effective components in honey and have a detrimental effect on human health. Herein, laser-induced breakdown spectroscopy (LIBS) combined with chemometric methods was used to fast quantify the adulterant content. Two common types of adulteration, including mixing acacia honey with high fructose corn syrup (HFCS) and rape honey, were quantified with univariate analysis and partial least squares regression (PLSR). In addition, the variable importance was tested with univariable analysis and feature selection methods (genetic algorithm (GA), variable importance in projection (VIP), selectivity ratio (SR)). The results indicated that emissions from Mg II 279.58, 280.30 nm, Mg I 285.25 nm, Ca II 393.37, 396.89 nm, Ca I 422.70 nm, Na I 589.03, 589.64 nm, and K I 766.57, 769.97 nm had compact relationship with adulterant content. Best models for detecting the adulteration ratio of HFCS 55, HFCS 90, and rape honey were achieved by SR-PLSR, VIP-PLSR, and VIP-PLSR, with root-mean-square error (RMSE) of 8.9%, 8.2%, and 4.8%, respectively. This study provided a fast and simple approach for detecting honey adulteration.

## 1. Introduction

Food adulteration is an illegal activity of food production, which may threaten food quality and safety. On one hand, the nutritional value of food is limited because of the reduction of effective components in food. On the other hand, the adulterants may have a detrimental effect on human health. Several scandals concerning food adulteration have been reported around the world [1,2,3]. Honey is one of the most commonly adulterated foods because of its economical purpose and wide use. There are two main approaches for honey adulteration. One is to mix pure honey with sugar-based adulterants, and the other is to adulterate high-quality honey with inferior honey. These two cases will be explored in this study.

The adulterant usually has a similar constituent or characteristic with the pure honey, and it is hard to distinguish from the appearance. Several studies concerning honey adulteration detection have been reported. Amiry et al. [4] discriminated adulterated honey (mix pure honey with date syrup and invert sugar syrup) with linear discriminant analysis. Different parameters including color indices, rheological, physical, and chemical parameters were used as variables for discrimination. Physical and chemical parameters achieved the best results, with accuracy above 95%. The results highlighted the use of physical and chemical parameters to detect honey adulteration. In addition, Arroyo-Manzanares et al. [5] used gas chromatography-ion mobility spectrometry to detect sugar cane or corn syrup adulterated honey; seven out of nine commercial honeys were classified as adulterated samples. Traditionally, the chemical features of honey are detected with wet chemical analysis, which is time and labor consuming. Hence, several rapid analytical methods based on electronic and optical techniques were proposed by other researchers, e.g., electronic nose [6], electronic tongue [6], fluorescence spectroscopy [7], visible-near infrared spectroscopy [8,9]. The ‘fingerprint information’ of honey could be rapidly obtained by these sensors, and the adulterated honey could be distinguished with the help of chemometric methods. 

For its part, laser-induced breakdown spectroscopy (LIBS), which allows elemental analysis, may be useful for honey authenticity. The elemental information of honey can be obtained through analyzing the atomic emission spectroscopy from plasma which is induced by a laser. It has the advantages of fast detection, multi-elemental analysis, and environmentally friendly feature [10]. As a novel approach in food, it has been used for regional discrimination [11] and elemental detection [12,13,14]. Because LIBS spectrum often contains numerous variables, chemometric methods are usually used to figure out the useful information and establish models for food adulteration detection. Recently, LIBS was used to classify the botanical origins of honey, and detect rice syrup adulterated samples [15]. However, the adulterant content in honey should be further quantified. Herein, LIBS combined with partial least squares regression was used as an analytical tool for fast quantification of honey adulterant content.

In this study, acacia honey mixed with high fructose corn syrup (HFCS) and rape honey were analyzed by LIBS. The specific objectives were to: (1) analyze the LIBS spectral features of pure honey and adulterants; (2) determine the feature variables that are related to adulteration; (3) quantify the adulterant content with univariate and multivariate analysis.

## 2. Materials and Methods

### 2.1. Sample Preparation

Honey including acacia honey (Guanshengyuan Co., Ltd, Shanghai, China) and rape honey (Yaoquan Food Co., Ltd, Yunnan, China) were collected from main producers in China, and two kinds of HFCS with different fructose concentrations (F55 and F90) were purchased from markets. HFCS F55 contains 55% fructose, and HFCS F90 contains 90% fructose. In this case, acacia honey was considered as pure honey, and HFCS (F55 and F90) and rape honey were used as adulterants.

Honey adulteration was prepared by mixing the acacia honey with HFCS F55, HFCS F90, and rape honey. To establish models for quantifying adulterant content, acacia honey was adulterated with HFCS and rape honey at 21 different percentages (0%, 5%, 10%, 15%, 20%, 25%, 30%, 35,% 40%, 45%, 50%, 55%, 60%, 65%, 70%, 75%, 80%, 85%, 90%, 95%, and 100%). In addition, adulterated samples for external prediction were prepared at 13 different adulteration rates, i.e., 0%, 8%, 16%, 24%, 32%, 40%, 48%, 56%, 64%, 72%, 80%, 88%, and 96%. The adulteration rates of 0% and 100% indicated pure acacia honey and pure adulterant, respectively. All sample adulteration was performed in three replications, so there were 63 samples for calibration, and 39 samples for prediction. After mixing, all samples were kept in a water bath at 37 °C for 12 h to ensure homogeneity.

### 2.2. LIBS Measurement

A laboratory-assembled LIBS device was used for honey adulteration detection. The detailed description of the device was introduced in our previous published article [16]. First, 8 g of sample was added in 12-well plates and placed in a X-Y-Z moving stage. A pulse laser (Vlite 200, Beamtech, Beijing, China) operated at 532 nm was used to ablate the sample with energy of 80 mJ. Then, emission light from induced plasma was transferred into an Echelle spectrograph (ME 5000, Andor, Belfast, UK), and detected by an intensified charge coupled device (ICCD, DH334T-18F-03, Andor, Belfast, UK). To improve the signal-to-background ratio, the delay time, integral time, and relative gain of ICCD camera were set at 2 µs, 10 µs, and 26. Single shot scanning was performed in an ablation region of 10 mm × 10 mm with resolution of 1 mm. Hence, 100 successive spectra were collected for each sample, the spectra were averaged to minimize the sample inhomogeneity. Because of the advantages of LIBS, no sample preparation was needed, and the total detection time for one sample was less than two minutes.

### 2.3. Data Analysis

Because the peak in LIBS spectrum corresponds to the emission from a certain element or molecule band, the observed peak intensity was used as the variable for analysis. To establish a model for quantifying adulterant content, PLSR was used. In addition, several feature selection methods based on PLSR were used to determine the key LIBS emissions that related to the adulterant content.

PLSR is a commonly and widely used multivariate method for quantitative analysis. It projects the raw variables into new dimensions with the maximal variation, and regresses the first few new variables (latent variable, LV) with respond value [17]. In this case, the raw variables were peak intensities of main emissions, and the respond value was the adulterant content in honey. Before modeling, the auto scale preprocessing method, which used mean-centering followed by dividing each variable by the standard variation of the variable, was used to correct the scaling of each variable. Ten-folds random cross-validation was used to determine the number of LV, and prevent the overfitting. In addition, the straightforward implementation of a statistically inspired modification of the PLS (SIMPLS) algorithm was used to calculate the PLS model parameters [18].

Three feature selection methods including genetic algorithm (GA), variable importance in projection (VIP), and the selectivity ratio (SR) were used in this case. GA is a subset search algorithm that was inspired by biological evolution theory and natural selection [19]. The subset of relevant variables selected by GA is then fitted with PLSR to evaluate the performance, and determine the feature variables. Different from GA, the variable selection based on VIP and SR is carried out by using a threshold of some parameters from the PLSR model. VIP calculates the accumulation of PLS weights, and SR defines the ratio between explained variance and the unexplained variance in the PLS model. The larger values of VIP and SR, the greater contribution of the variable. For the criteria of variable selection, VIP follows the rule of ‘greater than one rule’, and SR follows the F-test (95%) criterion [20]. In this case, the variables with VIP value greater than 1 and SR value greater than 1.532 were selected as important variables.

After modeling, some measures should be used to evaluate the performance. In this case, model performance was evaluated with correlation coefficient (*r*) and root-mean-square error (RMSE). The *r* value measures the relationship between predicted adulterant content and actual value, and the RMSE value measures the predictive error. The larger the *r* value and the smaller the RMSE value, the better the model performance. All data analyses were carried out in the MATLAB (v2019b, The MathWorks Inc., Natick, MA, USA).

## 3. Results and Discussion

### 3.1. LIBS Spectral Characteristics

**Figure 1:**
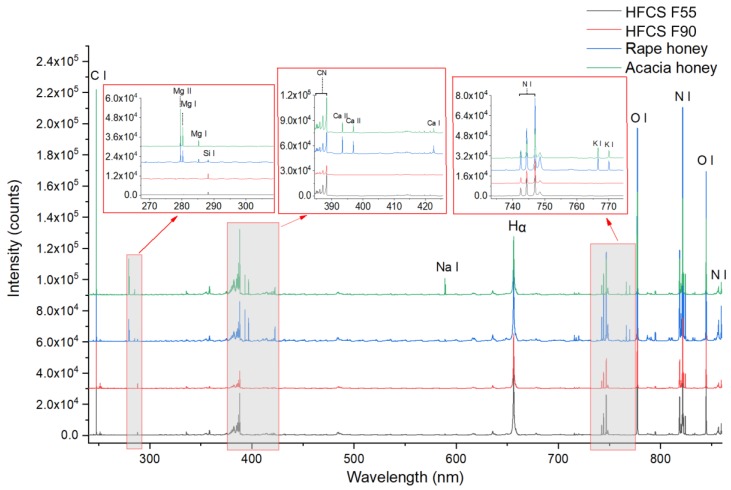
Average laser-induced breakdown spectroscopy (LIBS) spectrum of honey (acacia honey and rape honey) and high fructose corn syrup (HFCS 55 and HFCS 90).

Before quantification, LIBS spectral characteristics of acacia honey, rape honey, HFCS F55, and HFCS F90 were first analyzed (Figure 1). All the LIBS spectra ranged from 240 to 860 nm. In general, the average LIBS spectra for different samples were similar except some emissions in certain spectral range. It was credited to the similar constituent of honey and HFCS. In general, honey contains 75% saccharides (mainly glucose and fructose), 15% water, amino acids, and minerals, etc. HFCS mainly contains glucose and fructose. According to the concentration of fructose, the HFCS can be divided into three categories: F42 (42% fructose), F55 (55% fructose), and F90 (90% fructose). Hence, the main components ablated by laser in both honey and HFCS were glucose and fructose. As shown in Figure 1, the emissions from C, H, O, and N were observed in all samples. The molecular band CN that usually appears in an organic sample when analyzed in air atmosphere was also found in this case.

Some differences in elemental emissions could be observed between honey and HFCS. It was obvious that emissions from Mg, Ca, and K appeared in the spectra of honey, while it cannot be found in the spectra of HFCS. It indicated that the concentrations of Mg, Ca, and K in honey were significantly higher than those in HFCS. In addition, there was no obvious difference between acacia honey and rape honey, except relatively stronger emission of Na in acacia honey. These elemental differences might be used to differentiate the adulterants. However, it was hard to quantify the adulterant content simply by analyzing spectrum. Hence, some modeling methods were further used to quantify the adulterant content.

### 3.2. Univariate Analysis

Univariate analysis was used to explore the relationship between adulterant content and single variable and quantify the adulteration. In this case, the peak intensities of main emissions from samples were used for analysis. Univariate analysis was performed by regressing the peak intensity of each emission with the adulterant content, and *r* and RMSE were used to evaluate the results. The corresponding element for each emission could be identified with the National Institute of Standard and Technology (NIST, Gaithersburg, Maryland, USA) database [21]. Table 1 shows the results of univariate analysis between main emission lines and adulterant content. Forty-three univariate models were established. The variables contained emissions from C, Si, Mg, Ca, Na, K, N, H, O, and CN. Four variables with emissions of 748.47, 794.83, 795.17, and 822.43 nm were marked with unknown, because they could not be identified with the NIST database or references.

In general, the models for quantifying adulterant content of HFCS F90 had the best results with higher *r* and lower RMSE. It indicated that high concentration of fructose in HFCS led to greater spectral difference and contributed to the univariate analysis. In addition, for HFCS F90 and HFCS F55, the emissions from Mg II 279.58, 280.30 nm, Mg I 285.25 nm, Ca II 393.37, 396.89 nm, Ca I 422.70 nm, Na I 589.03, 589.63 nm, and K I 766.57, 769.97 nm had compact relationship with the adulterant content, with *r* > 0.9 and RMSE < 11.0%. For rape honey, models based on emissions from Na I 589.03 and 589.63 nm had good results, with *r* of 0.919 and 0.903, and RMSE of 12.0% and 13.0%. It indicated that emissions from mineral elements played an important role in adulteration quantification. It also verified the LIBS spectral difference between acacia honey and adulterants.

### 3.3. Quantification of Adulterant Content Based on Multivariate Analysis

Multivariate analysis was further used to quantify the adulterant content. First, all variables in univariate analysis were used as the inputs of PLS models. As seen in Table 2, PLS models based on all variables achieved good results for all three types of adulteration. The *r* values for HFCS F55, HFCS F90, rape honey in the prediction set were 0.962, 0.980, 0.988, and the RSME values were 15.6%, 16.6%, 4.7%, respectively. The latent variables for these three models were 4, 4, 5, which were determined by cross validation. The results of PLS models were better than those of univariate analysis. It also verified the advantages of multivariate analysis. The combination of information from multiple emissions contributed to the adulterant content quantification.

**Figure 2:**
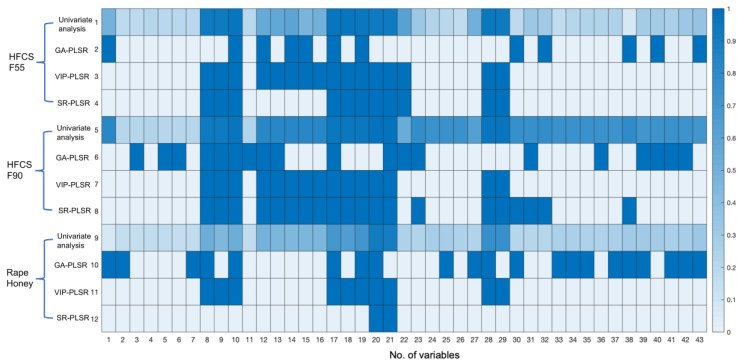
Feature variables selected with genetic algorithm (GA), variable importance in projection (VIP), and selectivity ratio (SR) methods. Row 1, 5, 9 shows the univariate analysis result between each variable and adulterant content of HFCS F55, HFCS F90, and rape honey, respectively. Cells with a gradient of blue color indicated the correlation coefficient. Other rows represented the variables selected by GA, VIP, and SR methods. Selected variables were represented in blue, and non-selected variables were in white.

In addition, results of PLS models based on feature variables (selected by GA, VIP, and SR) are also shown in Table 2. In general, prediction results after feature selection were similar or better than those based all variables. The irrelevant variables in models might worsen the modeling performance [22,23], which also verified the necessity of feature selection. Only one exception happened for the GA-PLS model in HFCS F55 quantification. The RMSE value in prediction set was 0.320, which is greatly worse than that without feature selection (0.156). It might be credited to the selected variables by the GA method. As shown in Figure 2, lots of irrelevant variables were selected. The GA method might not be suitable for feature selection in the honey adulteration with HFCS F55. With the consideration of variable number and prediction performance, the models marked with bold achieved the best results. The RMSE value for HFCS 55, HFCS F90, and rape honey in the prediction set were 8.9%, 8.2%, and 4.8%, respectively. In addition, similar results were achieved in 10-folds cross-validation, and RMSE value for HFCS 55, HFCS F90, and rape honey were 8.5%, 6.5%, and 4.6%, respectively.

We also compared the variables selected with GA, VIP, and SR methods (Figure 2). Row 1, 5, 9 showed the correlation coefficient between each variable and adulterant content of HFCS F55, HFCS F90, and rape honey, respectively. The values of correlation coefficient were in the range of 0 to 1. Other rows represented the variables selected by GA, VIP, and SR methods. Selected variables were represented in blue, and non-selected variables were in white. As shown in Figure 2, VIP and SR methods chose the variables with a high correlation coefficient, while some variables with a low correlation coefficient were selected by the GA method. It was related to the principal of feature selection methods. For the GA method, the variables were randomly combined and verified by PLSR. The variables were selected based on the results of PLSR modeling. For VIP and SR methods, the contribution of each variable was considered in the selection [20]. The variables selected by the GA method might be easily affected when testing with external samples. In addition, VIP and SR methods had some common variables, while the number of selected variables was different. It might be credited to the different threshold measure of each method. Hence, VIP and SR methods might be recommended for feature selection in quantification of honey adulterant content.

**Figure 3:**
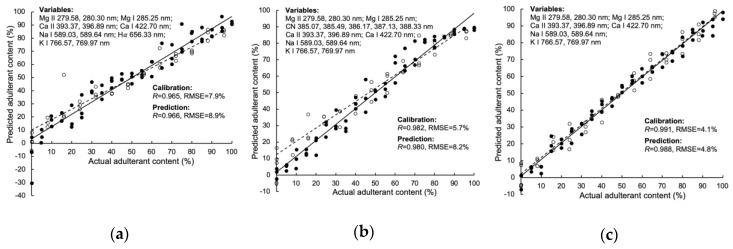
Scatter plot of actual adulterant content vs. LIBS measured adulterant content. Quantification of adulterant content in the mixture of (**a**) acacia honey and HFCS F55 based on the SR-PLSR model; (**b**) acacia honey and HFCS 90 based on the VIP-PLSR model; (**c**) acacia honey and rape honey based on the VIP-PLSR model.

The scatter plot of the best model for quantifying adulteration ratio of HFCS 55, HFCS 90, and rape honey is shown in Figure 3. Among these three models, the quantification for rape honey achieved the best result, with *r* and RMSE of 0.988 and 4.8% in the prediction set. The samples in calibration and prediction sets distributed closely around the regression lines, and the regression lines almost went through original point. The emissions from Mg II 279.58, 280.30 nm, Mg I 285.25 nm, Ca II 393.37, 396.89 nm, Ca I 422.70 nm, Na I 589.03, 589.64 nm, and K I 766.57, 769.97 nm, which were the feature variables in the rape honey quantification, were also included in the other two models. It indicated that these variables might play an important role in honey adulteration analysis.

## 4. Conclusions

In this study, LIBS combined with chemometric methods was used to detect honey adulteration. The adulterant content of acacia honey (adulterated with HFCS 55, HFCS 90, and rape honey) was successfully quantified. SR and VIP methods detected effectively the most relevant variables for adulteration determination. The emissions from Mg II 279.58, 280.30 nm, Mg I 285.25 nm, Ca II 393.37, 396.89 nm, Ca I 422.70 nm, Na I 589.03, 589.64 nm, and K I 766.57, 769.97 nm were considered as feature variables and played an important role in modeling. The importance of these variables was also verified in univariate analysis. The SR-PLSR, VIP-PLSR, and VIP-PLSR achieved the best results for detecting an adulteration ratio of HFCS F55, HFCS 90, and rape honey, with RMSE of 8.9%, 8.2%, and 4.8%, respectively. The results indicated the promising possibility of using LIBS and chemometric methods for quantification in honey adulteration. In addition, some research concerning model transfer could be explored, and more types of acacia honey as well as adulterants could be included in modeling in further study, which might be helpful for practical application. 

## Figures and Tables

**Table 1:** Results of univariate analysis based on peak intensities of main emissions.

No.	Observed Wavelength (nm)	Element	HFCS F55		HFCS F90		Rape Honey	
			*r*	RMSE	*r*	RMSE	*r*	RMSE
1	247.88	C I	0.493	26.4%	0.823	17.2%	0.066	30.2%
2	250.72	Si I	0.176	29.8%	0.204	29.6%	0.154	29.9%
3	251.45	Si I	0.180	29.8%	0.214	29.6%	0.153	29.9%
4	251.64	Si I	0.204	29.7%	0.222	29.5%	0.166	29.8%
5	251.94	Si I	0.193	29.7%	0.205	29.6%	0.159	29.9%
6	252.44	Si I	0.195	29.7%	0.210	29.6%	0.149	29.9%
7	252.88	Si I	0.200	29.7%	0.210	29.6%	0.158	29.9%
8	279.58	Mg II	0.932	10.9%	0.936	10.6%	0.516	25.9%
9	280.30	Mg II	0.922	11.7%	0.934	10.8%	0.441	27.2%
10	285.25	Mg I	0.959	8.6%	0.959	8.6%	0.517	25.9%
11	288.20	Si I	0.194	29.7%	0.227	29.5%	0.161	29.9%
12	385.07	CN 4-4	0.550	25.3%	0.828	17.0%	0.487	26.4%
13	385.49	CN 3-3	0.576	24.8%	0.820	17.3%	0.454	27.0%
14	386.17	CN 2-2	0.596	24.3%	0.821	17.3%	0.442	27.2%
15	387.13	CN 1-1	0.473	26.7%	0.828	17.0%	0.460	26.9%
16	388.33	CN 0-0	0.514	26.0%	0.824	17.2%	0.466	26.8%
17	393.37	Ca II	0.957	8.8%	0.948	9.6%	0.694	21.8%
18	396.89	Ca II	0.959	8.6%	0.951	9.3%	0.652	22.9%
19	422.70	Ca I	0.953	9.2%	0.942	10.1%	0.707	21.4%
20	589.03	Na I	0.937	10.6%	0.973	6.9%	0.919	12.0%
21	589.64	Na I	0.936	10.6%	0.975	6.8%	0.903	13.0%
22	656.33	Hα	0.617	23.8%	0.538	25.5%	0.243	29.4%
23	715.77	O I	0.316	28.7%	0.766	19.5%	0.227	29.5%
24	742.45	N I	0.220	29.5%	0.739	20.4%	0.268	29.2%
25	744.30	N I	0.197	29.7%	0.738	20.4%	0.278	29.1%
26	746.92	N I	0.162	29.9%	0.742	20.3%	0.248	29.3%
27	748.47	Unknown	0.507	26.1%	0.632	23.4%	0.220	29.5%
28	766.57	K I	0.943	10.1%	0.960	8.4%	0.756	19.8%
29	769.97	K I	0.931	11.1%	0.959	8.6%	0.750	20.0%
30	777.47	O I	0.183	29.8%	0.760	19.7%	0.215	29.6%
31	794.83	Unknown	0.316	28.7%	0.758	19.7%	0.215	29.6%
32	795.17	Unknown	0.299	28.9%	0.773	19.2%	0.206	29.6%
33	818.57	N I	0.170	29.9%	0.740	20.4%	0.256	29.3%
34	818.86	N I	0.217	29.6%	0.736	20.5%	0.265	29.2%
35	820.10	N I	0.232	29.5%	0.746	20.2%	0.237	29.4%
36	821.14	N I	0.221	29.5%	0.744	20.2%	0.247	29.3%
37	821.68	N I	0.244	29.4%	0.725	20.8%	0.250	29.3%
38	822.28	N I	0.067	30.2%	0.782	18.9%	0.305	28.8%
39	822.43	Unknown	0.290	29.0%	0.706	21.4%	0.303	28.8%
40	824.32	N I	0.291	29.0%	0.719	21.0%	0.285	29.0%
41	844.73	O I	0.252	29.3%	0.743	20.3%	0.260	29.2%
42	856.86	N I	0.325	28.7%	0.729	20.7%	0.275	29.1%
43	859.49	N I	0.357	28.3%	0.706	21.5%	0.316	28.7%

Note: The shade color of the table represents the performance of univariate analysis. The shade color of being green indicates the best compact relationship (*r* = ±1) and the lowest predictive error (RMSE = 0).

**Table 2:** Multivariate analysis results based on partial least square regression (PLSR) and feature selection methods.

Adulterant	Method	No. of LV	No. of Var.	Calibration		C.V.		Prediction	
				*r*	RMSE	*r*	RMSE	*r*	RMSE
HFCS F55	PLSR	4	43	0.977	6.5%	0.965	8.0%	0.962	15.6%
	GA-PLSR	4	12	0.983	5.6%	0.978	6.4%	0.794	32.0%
	VIP-PLSR	5	16	0.982	5.7%	0.966	8.1%	0.938	18.6%
	**SR-PLSR**	**1**	**11**	**0.965**	**7.9%**	**0.960**	**8.5%**	**0.966**	**8.9%**
HFCS F90	PLSR	4	43	0.973	7.0%	0.964	8.2%	0.980	16.6%
	GA-PLSR	5	19	0.979	6.1%	0.972	7.3%	0.985	11.3%
	**VIP-PLSR**	**5**	**15**	**0.982**	**5.7%**	**0.977**	**6.5%**	**0.980**	**8.2%**
	SR-PLSR	5	20	0.981	5.9%	0.973	7.0%	0.982	9.4%
Rape honey	PLSR	5	43	0.993	3.6%	0.990	4.3%	0.988	4.7%
	GA-PLSR	4	21	0.994	3.3%	0.990	4.4%	0.988	4.7%
	**VIP-PLSR**	**3**	**10**	**0.991**	**4.1%**	**0.989**	**4.6%**	**0.988**	**4.8%**
	SR-PLSR	1	2	0.912	12.4%	0.874	15.0%	0.943	11.3%

Note: No. of LV: number of latent variables; No. of var.: number of variables; C.V.: cross-validation; *r*: correlation coefficient; RMSE: root-mean-square error; GA: genetic algorithm; VIP: variable importance in projection; SR: selectivity ratio.
